# Dynamic shifts in plant-microbe relationships

**DOI:** 10.5511/plantbiotechnology.25.0428a

**Published:** 2025-09-25

**Authors:** Ivie Sonia Osayande, Xiaowei Han, Kenichi Tsuda

**Affiliations:** 1 National Key Laboratory of Agricultural Microbiology, Hubei Hongshan Laboratory, Hubei Key Laboratory of Plant Pathology, College of Plant Science and Technology, Huazhong Agricultural University, Wuhan 430070, China

**Keywords:** agricultural and ecosystem sustainability, host-microbe dynamics, interaction shift, microbe-microbe interactions, plant microbiome

## Abstract

Plant-microbe interactions encompass a continuum from mutualism and commensalism to parasitism. Mutualists confer benefits such as nutrient acquisition or stress tolerance, whereas pathogens compromise host health, and commensals coexist without detectable harm or benefit. Importantly, these relationships are not fixed but are dynamic, shifting between interaction modes in response to host physiology, microbial adaptation, and environmental conditions. Such shifts can influence plant health, agricultural productivity, and ecosystem stability. This review synthesizes the mechanisms underlying these functional transitions and discusses how understanding the drivers of interaction shifts can inform sustainable agriculture and ecosystem management.

## Introduction

Plant-microbe interactions exist along a functional spectrum comprising mutualism, commensalism, and parasitism (Hirsch 2004). Mutualistic microbes, such as nitrogen-fixing rhizobia in legumes or arbuscular mycorrhizal fungi (AMF) in many plant lineages, deliver critical services—ranging from nutrient acquisition to enhanced stress resilience—in exchange for plant-derived carbon (Oldroyd 2013; Wang et al. 2017). Commensals, by contrast, colonize plant tissues without imposing detectable costs or delivering obvious benefits (Zhang and Kong 2022). Pathogens reduce host fitness through active colonization and virulence mechanisms (Chen et al. 2021; Malmstrom et al. 2022; Wang et al. 2022).

**Figure 1:**
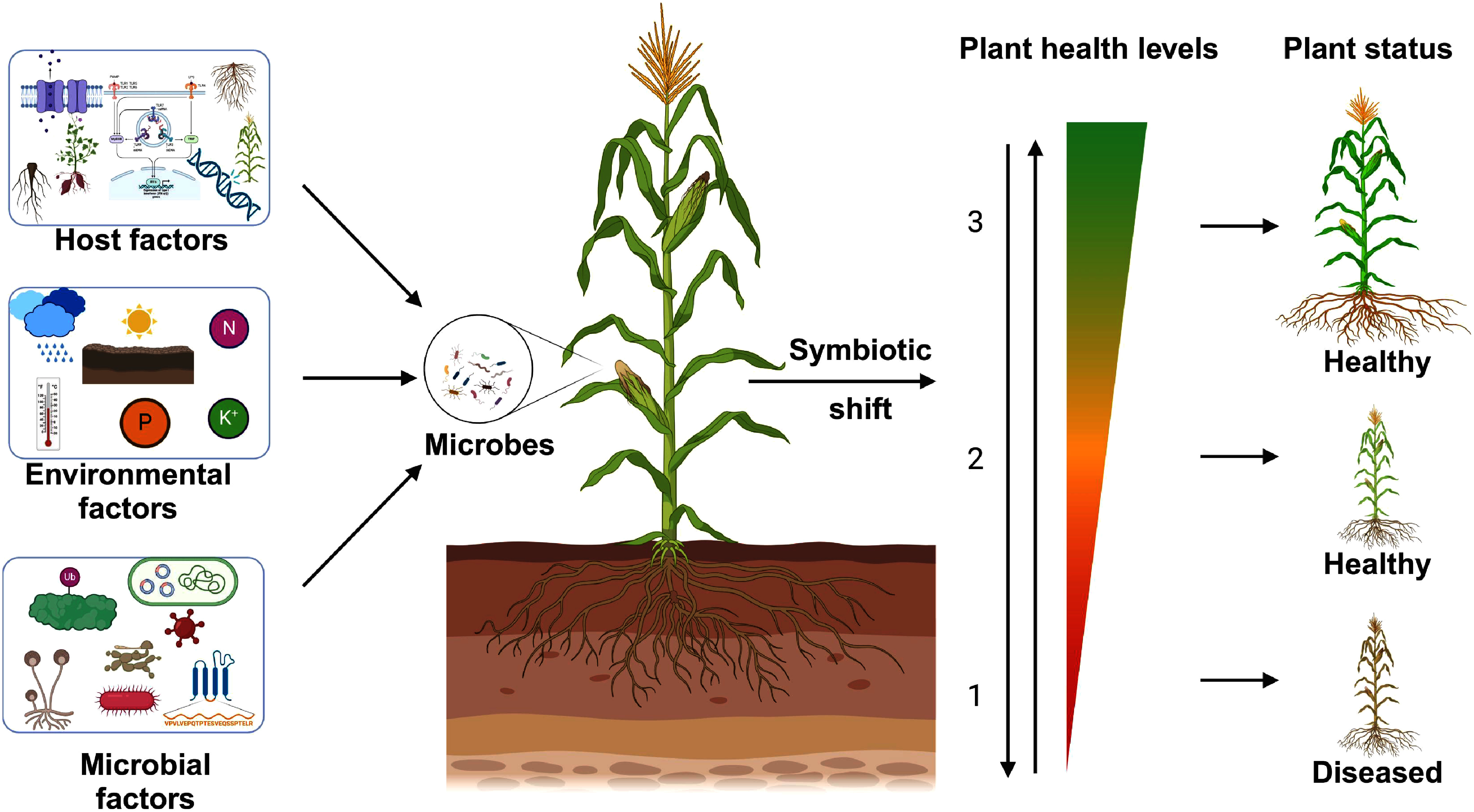
Figure 1. Multifactorial determinants of host-microbe symbiotic outcomes. Dynamic interaction shifts between mutualism, commensalism, and parasitism in plant-microbe interactions are governed by host and microbial factors, microbial community dynamics, and environmental conditions. These shifts lead to varying outcomes for plants, ranging from enhanced growth and health (mutualism) to neutral effects (commensalism) or disease development (parasitism). Figures were generated using Biorender [http://app.biorender.com].

Importantly, these microbial roles are not static. A microbe may function as a mutualist in one host, but act as a pathogen or commensal in other hosts. For example, host immune status can determine whether a microbe behaves benignly or causes disease. This review focuses on such *context-dependent transitions* (Figure 1), where dynamic shifts between mutualism, commensalism, and parasitism are driven by environmental factors, host physiology, or microbial community interactions, rather than fixed genetic determinants of compatibility. While canonical models of plant-pathogen interactions often emphasize binary outcomes (e.g., avirulent/virulent states defined by gene-for-gene recognition), this review highlights the plasticity of microbial lifestyles and the multifactorial drivers underlying their ecological roles. Understanding these dynamics is essential for developing sustainable approaches to agriculture and ecosystem resilience.

## Host plant determinants driving dynamic interaction shifts

Tryptophan-derived specialized metabolites play a pivotal role in modulating plant-microbe relationships, with their essential function in driving interaction shifts most prominently observed in *Arabidopsis thaliana*. For instance, the root fungal endophyte (lives inside an organism without causing harm) *Colletotrichum tofieldiae* exhibits context-dependent behavior, shifting between mutualism and parasitism based on host metabolic status. Under phosphate (Pi) limitation, *C. tofieldiae* promotes *A. thaliana* growth by directly supplying phosphorus. However, disruption of tryptophan-derived metabolites—particularly indole glucosinolates (IGS)—converts this mutualism into parasitism, underscoring their essential role in regulating fungal behavior (Hiruma et al. 2016). Host specificity within Brassicaceae further supports this mechanism. *Cardamine hirsuta*, a camalexin-deficient but IGS-retaining relative of *A. thaliana*, maintains mutualism with *C. tofieldiae*, whereas IGS-deficient *Capsella rubella* suffers growth suppression upon colonization, highlighting the importance of IGS in sustaining a beneficial association. In *A. thaliana*, mutants deficient in IGS biosynthesis (e.g., *cyp79b2b3*) exhibit hyper-colonization by *C. tofieldiae*, a phenotype also observed in interactions with beneficial fungi such as *Serendipita indica* and *Sebacina vermifera* (Lahrmann et al. 2015; Nongbri et al. 2012). IGS-derived metabolites, such as PEN2-dependent antifungal compounds, restrict fungal proliferation by activating plant defense pathways (Lahrmann et al. 2015; Nongbri et al. 2012). Collectively, these findings establish tryptophan-derived metabolites as key regulators of microbial lifestyle transitions, preventing pathogenic shifts across diverse plant-fungal systems.

Plant immune responses also determine microbial behavior. The bacterial strains *Xanthomonas* Leaf131 and Leaf148, originally isolated from healthy *A. thaliana* leaves, exhibit pathogenicity in plants lacking RBOHD, an NADPH oxidase required for the production of reactive oxygen species (ROS) during immune responses (Entila et al. 2024; Pfeilmeier et al. 2021). Mechanistically, RBOHD-generated ROS suppresses *Xanthomonas* virulence by downregulating the type II secretion system (T2SS). In wild-type plants, ROS inhibits the expression of *gspE*, a key T2SS component, thereby limiting the secretion of cell wall-degrading enzymes (CAZymes) and suppressing the pathogenic activity of *Xanthomonas* L148. In contrast, in *rbohD* mutant plants, unrestrained T2SS activity leads to pathogenic behavior (Entila et al. 2024). ROS-tamed *Xanthomonas* Leaf148 even confers protection against the foliar bacterial pathogen *Pseudomonas syringae*, illustrating how plant immunity can transform potentially pathogenic microbes into beneficial ones (Entila et al. 2024).

Dynamic shifts in plant-microbe interactions are not limited to individual microbial species but can also occur at the level of microbial communities. For instance, a synthetic community comprising bacteria, fungi, and oomycetes isolated from healthy *A. thaliana* has been shown to collectively promote *A. thaliana* growth (Durán et al. 2018). However, this beneficial effect is reversed in *A. thaliana* plants lacking tryptophan-derived specialized metabolites, resulting in fungal dysbiosis and adverse effects on plant health (Wolinska et al. 2021). These findings highlight the crucial role of tryptophan-derived specialized metabolites in modulating interactions within complex microbial consortia. Nonetheless, it remains unclear whether such community-level changes are driven by shifts in specific microbial traits or broader alterations in community dynamics. In summary, host-derived factors—including specialized metabolites and immune responses—play central roles in determining host-microbe relationships.

## Microbial determinants driving dynamic interaction shifts

Fungal secondary metabolism plays a pivotal role in modulating microbial behavior, enabling transitions between pathogenic and mutualistic states. Abscisic acid (ABA), well-documented for its role in promoting pathogen susceptibility across plant systems, critically governs symbiotic outcomes in *Colletotrichum tofieldiae* interactions. ABA facilitates pathogen infection by suppressing salicylic acid (SA)-mediated defenses and modulating host stress responses, a mechanism co-opted by pathogens like *Xanthomonas oryzae* and *Magnaporthe oryzae* to enhance virulence (Xu et al. 2013). In the pathogenic *C. tofieldiae* Ct3, sesquiterpene metabolites produced by a specific gene cluster activate the host ABA signaling pathway, promoting disease. However, disruption of this cluster converts pathogenic Ct3 into a growth-promoting mutualist (Hiruma et al. 2023). Recent work by Ujimatsu et al. further elucidates how dynamic shifts in *C. tofieldiae* behavior are mediated by the transcription factor CtBOT6. Overexpression of the transcription factor CtBOT6 activates the ABA-BOT cluster, enabling the beneficial Ct4 strain to adopt a pathogenic lifestyle in roots and leaves, while also suppressing host defenses through ABA-mediated mechanisms. This highlights the dual role of fungal secondary metabolites and transcriptional regulators in reprogramming host interactions, where CtBOT6 expression levels act as a molecular switch between mutualism and pathogenicity (Ujimatsu et al. 2025). Similarly, comparative genomic analyses between the pathogenic *Colletotrichum incanum*—a foliar pathogen of monocots and some *A. thaliana* accessions (Gan et al. 2016; Yang et al. 2014) and can be a root pathogen in *A. thaliana* (Hacquard et al. 2016)—and the beneficial *C. tofieldiae* reveal that *C. tofieldiae* retains many genes associated with pathogenicity and saprotrophy. This suggests that a functional shift rather than a structural genomic reorganization determines microbial behavior (Hacquard et al. 2016). These findings also suggest that preserving latent pathogenic potential may confer an evolutionary advantage, allowing *Colletotrichum* species to rapidly adapt to environmental changes and host availability.

Horizontal gene transfer also facilitates rapid microbial adaptation by enabling the acquisition of genetic elements that influence their functional roles during infection, often through virulence plasmids. In the bacterial genus *Rhodococcus*, strains can transition from beneficial to pathogenic upon acquiring a virulence plasmid, and revert to a mutualistic state when the plasmid is lost (Savory et al. 2017). These plasmids are essential for causing leafy gall disease in plants, although only a small subset of plasmid-encoded genes appears necessary for pathogenicity. The *fas* locus, which encodes cytokinin biosynthesis genes, is critical for virulence, yet the specific functions of individual *fas* genes remain unclear due to challenges in distinguishing microbial-derived cytokinins from those produced by the host during infection (Savory et al. 2017). Additionally, recombination events mediated by the *att* locus can convert non-virulence plasmids into virulence plasmids, accelerating the diversification of pathogenic *Rhodococcus* populations (Savory et al. 2017).

Phylogenomic analyses of *P. syringae* also illustrate transitions from commensals or mutualists to pathogens. Although best known as a plant pathogen, many phylogenetically related isolates function as commensals (Xin et al. 2018). The acquisition of the tripartite pathogenicity island (T-PAI), which includes the type III secretion system (T3SS) and associated effector genes, marks a major evolutionary step in the emergence of pathogenic *P. syringae* lineages. Conversely, early-branching lineages that lack the T3SS and its effectors exhibit commensal or mutualistic behavior (Xin et al. 2018). Understanding these mechanisms offers a path toward predicting and manipulating plant-microbe interactions.

Spontaneous mutations also drive behavioral shifts. Experimental evolution studies have demonstrated how pathogenic microbes can evolve into mutualists under selective pressure. For instance, the pathogenic bacterium *Pseudomonas protegens* CHA0 evolved into a plant growth-promoting mutualist within the rhizosphere of *A. thaliana* during a six-month association period (Li et al. 2021). This shift was driven by mutations in the *gacS*/*gacA* two-component regulatory system, known for regulating bacterial virulence. These mutations rendered *P. protegens* CHA0 mutualistic, conferring enhanced fitness, improved adaptation to root exudates, and reduced phytotoxicity compared to the ancestral strain (Li et al. 2021). This case underscores how subtle genetic changes can profoundly alter microbial behavior and influence the outcome of plant-microbe interactions.

## Microbe-microbe interactions as modulators of interaction shifts

Microbial interactions within communities can modulate functional outcomes. As mentioned above, *Xanthomonas* strains Leaf131 and Leaf148 are pathogenic to *A. thaliana* *rbohD* mutant plants but not in wild-type plants capable of producing ROS. In addition to ROS-mediated suppression, synthetic bacterial communities can also attenuate the virulence of these *Xanthomonas* strains, supporting the observation that they were originally isolated from healthy *A. thaliana* leaves (Bai et al. 2015; Entila et al. 2024; Pfeilmeier et al. 2021).

In another example, *Rhodococcus* Leaf278 confers protection against *P. syringae* within a synthetic bacterial community. This protective effect is more pronounced when Leaf278 is included in the community than when it is applied alone or omitted from the community (Vogel et al. 2021). These findings suggest that community-level interactions either enhance the beneficial effects of Leaf278 or that Leaf278 facilitates the protective function of the community as a whole.

Mycoviruses infection can also drive shifts in plant-fungal interactions by modulating fungal pathogenicity. For instance, the necrotrophic fungal pathogen *Sclerotinia sclerotiorum*, a major cause of stem rot in crops such as rapeseed, undergoes a dramatic behavioral shift upon infection with the mycovirus SsHADV-1. The mycovirus-infected strain DT-8 transitions from a virulent pathogen to an endophyte, characterized by the downregulation of plant cell wall-degrading enzymes and effector-like genes, along with alterations in hyphal development and growth rates (Tian et al. 2020; Zhang et al. 2020). This transition not only reduces fungal virulence but also promotes plant growth and enhances resistance to other fungal pathogens in rapeseed (Zhang et al. 2020). A similar transition occurs in the gray blight fungal pathogen *Pestalotiopsis theae*, where infection by Pestalotiopsis theae chrysovirus-1 converts the fungus into an endophyte in tea plants (Liu et al. 2022). Likewise, Stemphylium lycopersici alternavirus 1 (SlAV1) reduces the virulence of the necrotrophic fungal pathogen *Stemphylium lycopersici* by downregulating polyketide synthase genes responsible for the biosynthesis of Altersolanol A, a key phytotoxin. Strains infected with SlAV1 or engineered to express the viral ORF3 gene lose virulence and can even enhance plant resistance when applied as biocontrol agents (Liu et al. 2022; Zhou et al. 2021). These examples highlight the capacity of mycovirus to modulate fungal behavior, driving interaction shifts and converting virulent pathogens into commensal or even beneficial microbes.

At the community level, microbe-microbe interactions can similarly mediate functional shifts. A synthetic microbial community composed of bacteria, fungi, and oomycetes isolated from healthy *A. thaliana* promotes plant growth most effectively when all microbial groups are present (Durán et al. 2018). In contrast, synthetic communities composed solely of fungi, oomycetes, or their combination without bacteria exert detrimental effects and can even be lethal to plants. These findings underscore the essential role of bacterial members in converting potentially harmful microbial consortia into beneficial ones. Moreover, they suggest that individual microbes that are detrimental in isolation may exert positive effects within a balanced community, emphasizing the importance of maintaining microbial diversity and structural equilibrium for optimal plant health.

## Environmental determinants driving dynamic interaction shifts

Environmental factors critically influence plant-microbe interactions, driving bidirectional shifts in microbial behavior along the mutualism–parasitism continuum. For instance, *Pseudomonas brassicacearum* Root401, a commensal bacterium in *A. thaliana* under normal conditions, shifts to a pathogen under high salinity via a *brp* gene cluster-dependent mechanism (Getzke et al. 2024). Elevated salt levels induce the synthesis of brassicapeptin, a pore-forming lipopeptide encoded by the *brp* cluster. Brassicapeptin disrupts root cell membrane integrity, causing ion and nutrient leakage that exacerbates salt-induced osmotic stress (Getzke et al. 2024).

Similarly, the thermotolerant, plant growth-promoting rhizobacterium *Pseudomonas putida* AKMP7 enhances wheat tolerance to high temperatures under well-watered conditions (Shah et al. 2017). However, under drought stress, AKMP7 transitions from a mutualist to a pathogen due to dysregulated production of indole-3-acetic acid (IAA), a major developmental phytohormone. During drought, AKMP7 overproduces IAA, disrupting root water uptake and interfering with ABA-mediated stress responses. This auxin surge likely suppresses plant drought tolerance genes while prioritizing bacterial resource acquisition, a shift potentially mediated by stress-responsive regulators like RpoS or GacS/GacA. Notably, this pathogenicity is observed in *A. thaliana* but not in wheat, highlighting host-specific susceptibility to IAA-mediated sensitization and underscoring the need to elucidate auxin-ABA crosstalk in environmentally driven interaction shifts (Shah et al. 2017).

Light intensity is another determinant of interaction outcomes. *Diplodia mutila*, an asymptomatic fungal endophyte in *Iriartea deltoidea* palms under shaded conditions, becomes pathogenic under high light intensity through light-triggered metabolic reprogramming (Álvarez-Loayza et al. 2011). Increased light activates the fungal glyoxylate cycle, upregulating the expression of isocitrate lyase and malate synthase genes, which drives hydrogen peroxide production. This hydrogen peroxide synergizes with host-derived ROS to induce plant hypersensitive cell death. Concurrently, high light stimulates melanin biosynthesis via the polyketide synthase (PKS) pathway, enhancing fungal oxidative stress tolerance and virulence. Melanized hyphae secrete effectors that suppress plant immunity, while light-responsive transcription factors induce pycnidia formation, facilitating pathogenic spread. This dual strategy—ROS-driven host cell death and melanin-mediated fungal protection—underpins *D. mutila*’s niche-dependent behavior, limiting seedling survival in high-light environments while conferring herbivore resistance in shaded understories (Álvarez-Loayza et al. 2011; Mittler et al. 1997).

Temperature and atmospheric CO_2_ levels also drive functional transitions. The fungus *Aspergillus flavus* promotes sunflower growth under normal conditions. However, *A. flavus* shifts from a mutualist to a pathogen under elevated CO_2_ and temperature through climate-triggered aflatoxin biosynthesis and host immune sabotage (Maia et al. 2024). Elevated temperatures activate the fungal transcription factor AflR, upregulating the *aflS* aflatoxin cluster. Concurrently, elevated CO_2_ boosts carbohydrate metabolism, fueling aflatoxin production. Elevated temperatures suppress sunflower defenses by inhibiting SA synthesis and overloading chloroplasts with ROS via impaired Photosystem II electron transport. Fungal NADPH oxidase activity further exacerbates oxidative damage, while aflatoxin disrupts membrane integrity, compounding photosynthetic impairment (Maia et al. 2024).

Nutrient availability provides further evidence for environmentally driven interaction shifts. In *C. tofieldiae* Ct3, the outcome of interaction with *A. thaliana* is modulated by Pi availability (Hiruma et al. 2023). Under Pi-sufficient conditions, Ct3 behaves pathogenically, activating the host ABA pathway, which suppresses nutrient uptake. However, under Pi-limiting conditions, Ct3 exhibits attenuated pathogenicity, with reduced growth inhibition and partial contribution to Pi acquisition. This nutrient-dependent shift is regulated by the host Pi starvation response regulators PHR1 and PHL1. In contrast, *C. tofieldiae* Ct61 behaves as a root commensal under Pi-sufficient conditions but transitions to a mutualist under Pi limitation by actively supplying phosphate and promoting plant growth (Hiruma et al. 2016). These examples illustrate how environmental cues modulate the parasitism-mutualism continuum in plant-fungal interactions.

## Agricultural implications of interaction shifts

**Figure 2:**
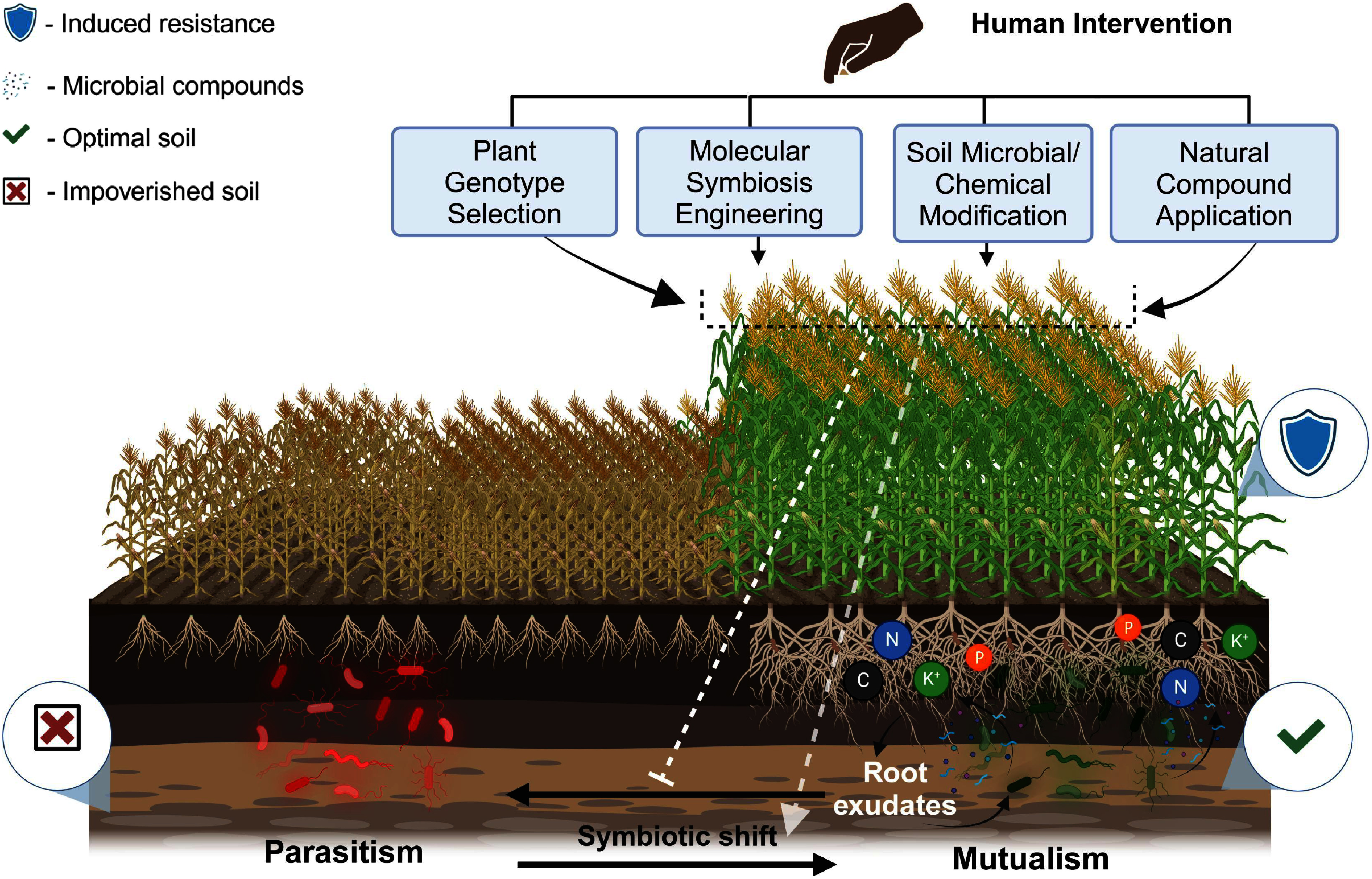
Figure 2. Strategies for optimizing dynamic interaction shifts in agriculture. Optimizing dynamic interaction shifts in agriculture can be achieved through the selection of plant genotype, modification of soil chemical properties, application of natural compounds, and manipulation of soil microbial community composition. These strategies help enhance crop yield and quality by promoting beneficial interactions. Figures were generated using Biorender [http://app.biorender.com].

Beyond the well-established roles of plant growth-promoting rhizobacteria and AMF as biofertilizers (Abkenar et al. 2024), dynamic shifts in plant-microbe interactions—driven by host factors, microbial adaptation, and environmental conditions—offer potential for sustainable agriculture. Understanding these transitions, where mutualists, commensals, and pathogens redefine their roles, is critical for developing strategies that harness or prevent such shifts to improve crop health and productivity. For instance, the pathogenic bacterium *Ralstonia solanacearum* exhibits nitrogen-responsive plasticity: it becomes virulent under nitrogen-rich conditions but remains benign in nitrogen-limited soils (Dalsing et al. 2015). This conditional behavior suggests that managing soil nitrogen levels could suppress parasitic transitions, maintaining a commensal state and reducing disease pressure (Figure 2).

Host genotype selection offers another avenue for directing favorable microbial shifts. In wheat, overexpression of the chitin receptor CERK1 confers resistance to fungal pathogens such as *Fusarium graminearum*, while maintaining compatibility with beneficial AMF colonization (Wang et al. 2024). Molecular breeding or genome editing that enhances receptor specificity could “lock in” mutualistic relationships and prevent opportunistic microbes from shifting toward pathogenicity.

Emerging tools like machine learning and microbiome monitoring further refine our ability to predict and direct dynamic shifts in plant-microbe relationships. Artificial intelligence models can analyze soil microbiome profiles to detect early signs of environmental imbalances—such as shifts in pH or nutrient availability—that may trigger a transition to pathogenicity (Pace et al. 2025). For example, real-time detection of quorum-sensing molecules from *Pectobacterium carotovorum* could prompt immediate countermeasures such as soil acidification to block virulence activation (Saha et al. 2015), showcasing how predictive analytics enable proactive microbial management.

Looking forward, the identification and manipulation of “interaction shift switch” genes—key molecular nodes that govern transitions between mutualism and parasitism—will be central to agricultural innovation. LysM receptors, which distinguish symbiotic lipo-chitooligosaccharides from pathogenic chitin fragments (Tan et al. 2025), represent promising targets for CRISPR-mediated editing in crops. Engineering receptor specificity could reduce the risk of beneficial microbes, such as AMF, inadvertently increasing susceptibility to oomycete pathogens (Wang et al. 2012).

While leveraging the plasticity of plant-microbe interactions offers promise for enhancing crop yields and reducing agrochemical inputs, it is equally important to weigh these interventions against long-term ecological consequences. Therefore, conceptualizing plant-microbe relationships as a dynamic continuum rather than fixed categories provides a roadmap for harmonizing agricultural productivity with ecosystem resilience. As mentioned above, potentially pathogenic microbial strains can be beneficial in the complex community context. By investing in interdisciplinary research that integrates molecular mechanisms, ecological context, and predictive modeling, we can develop innovative practices that secure the role of microbial partnerships as pillars of sustainable food systems and planetary health in a changing climate.

## Abbreviations

ABA: abscisic acid
CAZymes: carbohydrate-active enzymes
HGT: horizontal gene transfer
Pi: inorganic phosphate
SlAV1: Stemphylium lycopersici alternavirus 1
T2SS: type II secretion system
